# Identification and Characterization of VNI/VNII and Novel VNII/VNIV Hybrids and Impact of Hybridization on Virulence and Antifungal Susceptibility Within the *C*. *neoformans/C*. *gattii* Species Complex

**DOI:** 10.1371/journal.pone.0163955

**Published:** 2016-10-20

**Authors:** Mojgan Aminnejad, Massimo Cogliati, Shuyao Duan, Michael Arabatzis, Kathrin Tintelnot, Elizabeth Castañeda, Marcia Lazéra, Aristea Velegraki, David Ellis, Tania C. Sorrell, Wieland Meyer

**Affiliations:** 1 Molecular Mycology Research Laboratory, Centre for Infectious Diseases and Microbiology, Sydney Medical School – Westmead Hospital, Marie Bashir Institute for Infectious Diseases and Biosecurity, The University of Sydney, Westmead Institute for Medical Research, Sydney, Australia; 2 Laboratory Micologia Medica, Dip. Scienze Biomediche per la Salute, Università degli Studi di Milano, Milano, Italy; 3 Mycology Research Laboratory, Department of Microbiology, Medical School, National Kapodistrian University of Athens, Athens, Greece; 4 Mycology Group, Robert Koch Institute Berlin, Berlin, Germany; 5 Grupo de Microbiología, Instituto Nacional de Salud, Bogotá, Colombia; 6 Mycology Laboratory, National Institute of Infectious Diseases, Oswaldo Cruz Foundation, Rio de Janeiro, Brazil; 7 School of Molecular & Biomedical Sciences, University of Adelaide, Adelaide, SA 5005, Australia; University of Minnesota, UNITED STATES

## Abstract

*Cryptococcus neoformans* and *C*. *gattii* are pathogenic basidiomycetous yeasts and the commonest cause of fungal infection of the central nervous system. Cryptococci are typically haploid but several inter-species, inter-varietal and intra-varietal hybrids have been reported. It has a bipolar mating system with sexual reproduction occurring normally between two individuals with opposite mating types, α and **a**. This study set out to characterize hybrid isolates within the *C*. *neoformans*/*C*. *gattii* species complex: seven unisexual mating intra-varietal VNI/VNII (αAAα) and six novel inter-varietal VNII/VNIV (**a**ADα). The *URA5*-RFLP pattern for VNII/VNIV (**a**ADα) differs from the VNIII (αAD**a**) hybrids. Analysis of the allelic patterns of selected genes for AD hybrids showed 79% or more heterozygosis for the studied loci except for CBS132 (VNIII), which showed 50% of heterozygosity. MALDI-TOF MS was applied to hybrids belonging to different sero/mating type allelic patterns. All hybrid isolates were identified as belonging to the same hybrid group with identification scores ranging between 2.101 to 2.634. All hybrids were virulent when tested in the *Galleria mellonella* (wax moth) model, except for VNII/VNIV (**a**ADα) hybrids. VNI/VGII hybrids were the most virulent hybrids. Hybrids recovered from larvae manifested a significant increase in capsule and total cell size and produced a low proportion (5–10%) of giant cells compared with the haploid control strains. All strains expressed the major virulence factors—capsule, melanin and phospholipase B—and grew well at 37°C. The minimal inhibitory concentration of nine drugs was measured by micro-broth dilution and compared with published data on haploid strains. MICs were similar amongst hybrids and haploid parental strains. This is the first study reporting natural same sex αAAα intra-varietal VNI/VNII hybrids and **a**ADα inter-varietal VNII/VNIV hybrids.

## Introduction

The basidiomycetous yeasts *Cryptococcus neoformans* and *Cryptococcus gattii* are important human pathogens infecting mainly immunocompromised and immunocompetent individuals, respectively [1, 2]. For the purpose of this study the traditional two species and 8 molecular type classification is used [3]. *C*. *neoformans* is comprised of two varieties: *C*. *neoformans* var. *grubii* (serotype A; molecular types VNI and VNII) and *C*. *neoformans* var. *neoformans* (serotype D; molecular type VNIV) [4]. In addition, serotype AD hybrids (molecular type VNIII) which are either diploid or aneuploid, have been reported in clinical and environmental samples [4–12]. Multi-locus sequence typing (MLST) identified in addition a new molecular type (VNB) of serotype A, which was isolated in Botswana [13], Brazil [14], and Italy [15]. The sibling species *C*. *gattii* has four widely accepted molecular types (VGI, VGII, VGIII, VGIV) distributed amongst two serotypes (B and C) [4, 16]. Three inter-species hybrid strains were subsequently reported; two BD hybrids (*C*. *neoformans* var. *neoformans* VNIV × *C*. *gattii* VGI), from HIV-negative patients in the Netherlands and one AB hybrid (*C*. *neoformans* var. *grubii* VNI × *C*. *gattii* VGI), from an HIV-positive patient in Canada [17, 18]. More recently, three AB hybrids (*C*. *neoformans* var. *grubii* VNI × *C*. *gattii* VGII) from HIV-negative patients in Brazil and Colombia and one AB hybrid (*C*. *neoformans* var. *grubii* VNI × *C*. *gattii* VGI) from an HIV-positive patient in India have been described [19].

*C*. *neoformans* is typically haploid with a bipolar heterothallic mating system containing one mating type locus, which occurs in either of the two alleles, **a** or α. Sexual reproduction normally occurs between opposite mating types [20]. Serotype A is the predominant serotype, being responsible for over 90% of all cases in HIV-positive patients [21–23]. The fact that the mating type α is preponderant amongst environmental and clinical isolates of *Cryptococcus* regardless of the serotype [24] raises the question of how sexual reproduction might occur in this unisexual population. Same-sex mating (α-α) occurs in nature as evidenced by the existence of αADα [25] and αAAα (VNII/VNB) hybrids [26, 27]. Furthermore, population genetic studies detected sexual recombination in natural populations of both *C*. *neoformans* var. *grubii* and *C*. *gattii*, where only the mating type α was identified, including α-only populations of *C*. *neoformans* var. *grubii* veterinary isolates in Sydney, Australia [28], environmental *C*. *neoformans* var. *grubii* isolates from tree hollows in north-western India [29] and *C*. *gattii* isolates from Australian eucalyptus trees [30]. Phylogenetic analysis of the *C*. *gattii* outbreak strains from Vancouver Island speculated that same-sex mating between two different mating type α strains may have given rise to a more virulent strain which then occupied a new environmental niche [31].

Multiple recent hybridization events ranging from present time to 2.158 million years ago between strains of both serotypes A and D have been suggested to be responsible for the origin and current distribution of AD hybrid strains [12]. As such, they contain alleles of both serotypes and are diploid or aneuploid [10–12]. The clinical relevance of AD hybrids is emphasised by their presence in several parts of the world. A European study of 311 clinical cryptococcal isolates collected from 1997 to 2001, showed that 30% of the studied isolates were AD hybrids, with the highest incidence being observed in Portugal (50%), Greece (48%) and Spain (45%) [23]. An analysis of clinical and environmental *C*. *neoformans* isolates in the United States showed that 7.1% of the environmental and 2.4% of the clinical strains were AD hybrids [32]. Most serotype AD strains are heterozygous at the mating locus, presenting two opposite mating type alleles, αAD**a** or **a**ADα. Although, the *MAT****a*** allele among isolates of serotype A is rare, it is common in AD hybrids [9–11, 32]. Natural αADα hybrids that arose by fusion between two α cells of different serotypes have been reported in Southern Africa and the USA [25]. In addition, it has been reported that a small percentage of AD strains are homozygous at the mating locus, presenting only one mating type allele [23, 33].

*C*. *neoformans* and *C*. *gattii* have a set of well-defined virulence factors which influence pathogenicity of individual isolates, including melanisation, capsule production, phospholipase B secretion and ability to grow at body temperature. These virulence properties enable *C*. *neoformans* and *C*. *gattii* to be the only two highly successful pathogens in the genus *Cryptococcus* [20]. Several studies have shown that hybrids with different mating type allelic patterns differ in virulence [10, 34, 35]. Diploid progeny of serotype A and D have been reported to be more fit than their parental strains [36]. Isogenic hybrids showed that ploidy and mating type allelic patterns do not affect the virulence in a mouse model and that higher ploidy has a minor effect in reduction of virulence [37]. However, another study has shown that there is no correlation between mating type locus pattern and mortality in a mouse model [38]. The true incidence of clinical hybrid strains is unknown, as inter-varietal or inter-species hybrids are not routinely identified in diagnostic laboratories, which may explain the small number of intra-varietal hybrids reported to date [26, 27].

The current study set out to gain knowledge of the presence of hybrids in our large global collection of *C*. *neoformans*/*C*. *gattii* strains using molecular and proteomics approaches, and to obtain further insides into the genetic make up, antifungal susceptibility, virulence phenotypes, and pathogenicity of potential hybrids, to provide insights into the impact of hybridization.

## Materials and Methods

### Isolates and reference strains

A total of 365 global epidemiologically unlinked *C*. *neoformans* clinical (n = 172), environmental (n = 15) and veterinary (n = 170) isolates were selected from the culture collection of the Molecular Mycology Research Laboratory at Westmead Hospital, Australia and screened for the presence of potential hybrids. These isolates were from Australia (n = 202), South Africa (n = 33), Brazil (n = 31), Colombia (n = 16), Peru (n = 10), Italy (n = 9), Thailand (n = 9), USA (n = 9), New Zealand (n = 8), Argentina (n = 6), Belgium (n = 5), Chile (n = 3), India (n = 3), Canada (n = 2), Greece (n = 2), Japan (n = 2), Spain (n = 2), Germany (n = 1), Kuwait (n = 1), Mexico (n = 1), Venezuela (n = 1) and Zaire (n = 1). In addition, two hybrid strains from the Laboratory Micologia Medica, Dipartmento di Scienze Biomediche per la Salute, Universita degli studi di Milano, Milan, Italy were included in the study. Eight standard strains, representing each of the major molecular type of the *C*. *neoformans/C*. *gattii* species complex (WM 148 (VNI, Aα), WM 626 (VNII, Aα), WM 628 (VNIII, αAD**a**), WM 629 (VNIV, Dα), WM 179 (VGI, Bα), WM 178 (VGII, Bα), WM 175 (VGIII, Bα), WM 779 (VGIV, Cα)) were used for the identification of the major molecular types [4]. In addition, the following strains were included as references for specific tests, e.g. mating type, serotype, virulence phenotype and pathogenicity studies: H99 (VNI, Aα) [39], IUM 96–2828 (VNI, A**a**) [40], JEC21 (VNIV, Dα) [41], JEC20 (VNIV, D**a**) [41], CBS132 (VNIII, αAD**a**) [11, 42], IUM 91–0804 (VNIII, **a**ADα) [11], CDC R265 (VGIIa, Bα) [43] and CDC R272 (VGIIb, Bα) [44]. Four additional inter-species cryptococcal hybrids (WM 2617 (VNI/VGI, αAB**a**), WM 05.272 (VNI/VGII, αAB**a**), WM 05.459 (VNI/VGII, αAB**a**), WM 05.532 (VNI/VGII, αAB**a**)) described previously [19] were used in the MALDI-TOF MS, antifungal susceptibility and virulence analyses.

### Identification of hybrids

Genomic DNA of all isolates was extracted according to Ferrer *et al*. [45]. To determine the molecular type of the isolates, *URA*5-RFLP was first amplified with the primers URA5, 5'-ATGTCCTCCCAAGCCCTCGACTCCG-3' and SJ101, 5'-TTAAGACCTCTGAACACCGTACTC-3' and PCR products were digested with *Hha*I and *Sau*96I enzymes at 37°C over night [4]. The molecular types were identified by comparison of each of the obtained profiles with those of the eight standard strains of the eight major molecular types [4]. Isolates with mixed patterns were considered potential hybrids. Luminex analysis was performed on potential intra-varietal hybrid isolates using the oligonucleotide probes described by Diaz and Fell [46] and two probes specifically designed for the VNI and VNII molecular types [19] on the Luminex 100 analyzer (Luminex Corporation, Austin, TX, USA) as described previously by Diaz and Fell [46] and Diaz *et al*. [47], and median fluorescent intensity (MFI) values were calculated. The DNA content of hybrids was measured by flow cytometry as described previously [48]. Briefly, overnight grown cells were harvested from YPG (1% yeast extract, 1% pepton and 2% glucose) medium supplemented with 0.5 M sodium chloride and washed twice with distilled water. 10^7^ cells/ml were fixed in 1 ml of 70% ethanol overnight at 4°C. Fixed cells were washed with water and NS buffer (10 mM Tris-Hcl (pH 7.2), 250 mM sucrose, 1 mM disodium EDTA, 1 mM MgCl_2_, 0.1 mM ZnCl_2_) and then stained with 1 ml of propidium iodide (10 μg/ml) in NS buffer containing RNase A (1 mg/ml) at 37°C for 3–4 h before incubation at 4°C overnight. Then cells were washed and diluted in 1 ml of 15 mM Tris-HCl (pH 8.0) and sonicated for 1 min. Flow cytometry was performed on 10,000 cells for each potential hybrid isolate and analyzed with a Becton Dickinson FACSCallibur instrument (BD Biosciences, San Jose, CA, USA). Strain H99 (VNI, Aα) was used as haploid reference. Cells stained with propidium iodide were examined by fluorescent microscopy to check the number of nuclei per cell.

### Determination of mating type and serotype by PCR

The primer sets MFαF/MFαR and MFaF/MFaR [49], STE12αF809/STE12αR1607 and STE12aF537/STE12aR1299 [17] were used to determine the mating type of the hybrid isolates. To identify the serotype-specific allele combinations, the *CAP59* gene was amplified and the amplicons were digested using two separate restriction enzymes–*Bsm*FI and *Hpa*II as described previously [50]. For the intra-varietal hybrid isolates the presence or absence of serotype A- and D-specific alleles of the *GPA1* gene was determined by PCR using the primer set JOHE2596/JOHE3241 and JOHE2596/JOHE3240 [10] and for *PAK1* gene the primer set JOHE3066/JOHE3236 and JOHE3066/JOHE3065 D was used [25]. Moreover, several PCRs were performed on intra-varietal hybrids for the serotype- and mating-type-specific genes *STE20α/****a*** and *SXI1α/2****a***, with the primer sets JOHE7264/JOHE7266, JOHE7267/JOHE7269, JOHE7270/JOHE7271, JOHE7273/JOHE7274, JOHE15634/JOHE15635, JOHE15634/JOHE15636, JOHE15629/JOHE15630 and JOHE15629/JOHE15631 as published previously [25]. Primer sequences are listed in S1 Table.

### Determination of α/α diploidization mechanism

To determine the mechanism of α/α diploid formation, polymorphisms in two highly divergent loci located in the mating type locus (*SXI1α*, *STE20α*) and other polymorphic genomic regions (*GPD1*, *PLB1*, *SOD1*, *URA5* and IGS1) were examined after PCR amplification followed by sequencing. Primer sequences and amplification conditions are listed in S2 Table. If a diploid α/α isolate resulted by mating between two genetically distinct α cells, the isolate would show heterozygosis (nucleotide polymorphisms) at both the *MAT* locus and other genomic regions [26].

### Characterization of AD hybrids

To determine mating-type allelic pattern of AD hybrids a multiplex PCR was performed with four pairs of primers designed based on the nucleotide sequences of *NCP1a*, *NAD4α*, *STE20a* and *STE20α* mating type-specific genes as described previously [51]. To investigate the level of heterozygosity of each AD hybrid, allele specific PCRs were performed with serotype A- and D-specific primers based on the sequences of 14 loci (*IGS1*, *GPD1*, *SOD1*, *LAC1*, *PLB1*, *CAP59*, *CAP10*, *URE1*, *GPA1*, *PAK1*, *CNA1*, *LBP*, *MDT* and *CDK*) located on different chromosomes, as described previously [38]. The percentage of heterozygosity of each hybrid isolate was estimated by dividing the number of heterozygous loci by the total number of loci analyzed.

### Application of MALDI-TOF MS for identification of hybrid isolates

Test strains were grown on Sabouraud’s dextrose agar for 48 hr at 30°C. Protein extracts were prepared using the ethanol-formic acid extraction protocol adopted from Bruker Daltonics [52] as described previously [53]. Briefly, 1 μl of the protein extract was placed on the target position and overlaid with 1 μl of HCCA (α-cyano-4-hydroxy-cinnamic acid) matrix, (Bruker Daltonics). Each sample was applied to 6 different spots on the plate. After air-drying the samples at room temperature, measurements were performed with a Microflex LT mass spectrometer (Bruker Daltonics). Spectral processing and identification were performed using MALDI-Biotyper 3.0 software (Bruker Daltonics). For each tested isolate, using the MALDI Biotyper Automation Control software version 2.0.43.8 (Bruker Daltonics), a composite of 6 spectra was generated, resulting in a main spectrum (MSP), which contains the frequencies of the most significant peaks, average mass and intensity. The MSP of each isolate was used for pattern matching against the extended Biotyper 3.0 library database and our in-house MSP library entries [40], consisting of 20 *Cryptococcus* isolates for each major molecular type (160 MSP) and identification scores were generated using the Biotyper 3.0 software (Bruker Daltronics). Values of 2.300–3.000 are rated as a highly probable species identification, values of 2.000–2.299 are rated as secure genus identification with probable species identification.

### *G*. *mellonella* survival assay and recovery of cryptococcal cells

Similar sized (approximately 250 mg body weight) *G*. *mellonella* larvae (20 larvae per strain) were infected by injecting 10^6^ fungal cells into the hemocoel at the last left pro-leg with a 26 gauge Hamilton syringe [54]. All larvae within an infected group were then transferred to a 90 mm plastic Petri dish loosely covered with aluminium foil and incubated at 37°C in the dark. The larvae were observed daily for 14 days and the number of dead larvae was scored daily. Larvae were considered to be dead when they failed to respond to gentle poking with forceps. One group of control larvae was inoculated with PBS to ensure that death is not due to trauma and that the buffer has no impact on larval viability and another group of larvae was not inoculated to monitor the overall quality of the larvae during the course of the experiment. Killing curves were plotted by GraphPad Prism 6 (GraphPad Software, Inc., CA, USA) and the estimation of differences in survival was analysed by the Kaplan-Meier method using SPSS Statistics version 21.0 (IBM, NY, USA) statistical software. A *p*<0.05 was considered significant.

To recover cryptococcal cells from *G*. *mellonella*, larvae were mashed in 1 ml of PBS and filtered through a strainer with a pore size of 100 μm (BD Biosciences, San Jose, California, USA). Homogenates were washed twice with 1 ml of PBS and suspended in 150 μl of PBS. Cryptococci were suspended in India ink, observed by microscopy and photographed. To measure differences in capsule and total cell size pre- and post-infection, cryptococcal cells grown overnight in YPD broth were also observed by microscopy (20 cells for each isolate). Cell body (delimited by cell wall) and capsule sizes were measured using QCapture Pro software v. 5.0.1.26 (QImaging, Surrey, BC, Canada). Total cell size was defined as the diameter of each cell including the capsule. Capsule size was calculated as the difference in the diameter of the cell plus capsule and the cell body. All cell and capsule measurements were log transformed to approximate normality to establish the variance prior to analysis and paired t-tests were performed using SPSS Statistics version 21.0 (IBM, NY, USA) software to compare the mean change in ratio of capsule size to whole cell body size pre versus post inoculation. A Spearman rank correlation was performed using the SPSS Statistics version 21.0 (IBM, NY, USA) between median survival time and proportion of giant cells, median total cell size, median cell body size, median capsule size and median ratio of capsule to total cell size after recovering the cells from infected larvae for each tested isolate. Also, Spearman rank correlation was performed using SPSS Statistics version 21.0 (IBM, NY, USA) between median survival time and median total cell size, median cell body size, median capsule size and median ratio of capsule to total cell size before and after injection to the larvae for each hybrid group and the control strains.

### *In vitro* analysis of virulence factors

Hybrid and haploid control strains were grown overnight at 30°C in liquid YPD. Cells were pelleted by centrifugation. Serial 10-fold dilutions of the cells were prepared in saline to determine cell density by counting using a haemocytometer. To analyze growth at high temperature, cells were spotted on YPD agar and incubated at 37°C for 3 days. As a control, another set of plates was incubated at 30°C. Production of melanin was tested at 30°C on melanin-inducing L-DOPA agar medium [55, 56]. Melanization was identified by the development of dark brown colonies after 3 days. To characterize capsule induction, 10^6^ cells of each strain were cultured on capsule-inducing RPMI-1640 agar and incubated for 2 days at 35°C in the presence of 5% CO_2_ [57, 58]. Cells were suspended in India ink and capsules were visualized by light microscopy and measured using QCapture Pro v. 5.0.1.26 (QImaging, Surrey, BC, Canada). All capsule measurements were log-transformed to approximate normality to establish the variance prior to analysis. Analysis of variance (ANOVA) was performed using the SPSS Statistics software, version 21.0 (IBM, NY, USA). The mean ratio of capsule size to total cell size was compared for each group of hybrids. Phospholipase activity was tested by inoculating 10^6^ cells on Sabouraud Dextrose Agar (SDA) containing 1 M sodium chloride, 0.005 M calcium chloride and 8% sterile egg yolk (Micromedia Laboratories, Vic, Australia). The plates were incubated at 37°C for 7 days and the diameter of the zone of precipitation around the colonies was measured. The ratio of the diameter of the colony to the diameter of the colony plus precipitation zone (Pz) was measured as an index of phospholipase activity. A Pz value of 1.0 indicates that the test strain is phospholipase negative [59].

### *In vitro* antifungal susceptibility testing

Antifungal activity was determined by the Sensititre YeastOne YO9 Microdilution Test Panel (Trek Diagnostics, UK) which is a microtitre broth dilution method based on the CLSI M27-A3 standard [60]. Each test incorporates serial dilutions of amphotericin B, fluconazole, itraconazole, posaconazole, 5-flucytosine and voriconazole. Plates were read after 72 hr of incubation at 35°C. The MICs of each drug were log transformed prior to analysis.

## Results

### Identification and molecular characterization of hybrids

From the 365 isolates studied, 19 isolates were identified as hybrids including 4 inter-species isolates previously described [19], seven intra-varietal (VNI/VNII, αAAα) and eight inter-varietal (VNII/VNIV, aADα) hybrid isolates. The specific characteristics of the identified hybrid isolates is summarised in Table 1.

**Table 1:** Characteristics of the studied strains.

Strain #	Other collection #	Origin	Source	Year of isolation	Underlying disease	Site of Sample	Molecular Type	Mating/ Serotype	MALDI-TOF Identification	MALDI-TOF Score^c^	Phospholipase zone (Pz)	Reference
WM 714^a^	Liimatta	Australia	Veterinary	1994	FIV+	Paranasal	VNI/VNII	αAAα	VNI/VNII	2.540	0.73	This study
WM 1986	LA 511; HOO58 I-190	Bogota, Colombia	Clinical	1992	HIV+	CSF	VNI/VNII	αAAα	VNI/VNII	2.549	0.65	This study
WM 2059	LA 588; LA 227; HOO58 I-718	Bogota, Colombia	Clinical	1998	HIV+	CSF	VNI/VNII	αAAα	VNI/VNII	2.605	0.56	This study
WM 2064	LA 595; HOO58 I-828	Bogota, Colombia	Clinical	1998	HIV+	CSF	VNI/VNII	αAAα	VNI/VNII	2.605	0.61	This study
WM 2068	LA 602; HOO58 I-889	Bogota, Colombia	Clinical	1999	HIV+	CSF	VNI/VNII	αAAα	VNI/VNII	2.549	0.62	This study
WM 2975	PAH-15	Brisbane, Australia	Clinical	1994	HIV-	lung	VNI/VNII	αAAα	VNIII	2.287	0.65	This study
WM 05.269	HOO58-I-989	Medellin, Colombia	Clinical	1999	HIV+	CSF	VNI/VNII	αAAα	VNI/VNII	2.543	0.73	This study
WM 1693	HM 135610; Arg-C26	Buenos Aires, Argentina	Clinical	1998	HIV+	CSF	VNII/VNIV	aADα	VNII/VNIV	2.197	0.59	This study
WM 1876	IMIM 73[c]; Spa-E13	Barcelona, Spain	Clinical	ND	ND	ND	VNII/VNIV	aADα	VNII/VNIV	2.436	0.67	This study
WM 1877	IMIM 75 (c); Spa-E15	Barcelona, Spain	Clinical	ND	ND	ND	VNII/VNIV	aADα	VNII/VNIV	2.404	0.54	This study
WM 05.549	LMM 837	Salvador, Bahia, Brazil	Clinical	2001	HIV+	CSF	VNII/VNIV	aADα	VNII/VNIV	2.443	0.56	This study
WM 09.183	IFM 45751	Noujing, China	Environmental	ND	NA	Pigeon droppings	VNII/VNIV	aADα	VNII/VNIV	2.443	0.67	This study
WM 09.184	IFM 45759	Noujing, China	Environmental	ND	NA	Pigeon droppings	VNII/VNIV	aADα	VNII/VNIV	2.026	0.64	This study
-	IUM02-1102, UA2341^b^	Ancona, Italy	Clinical	1995	HIV+	CSF	VNII/VNIV	aADα	ND	ND	ND	[35, 38]
-	IUM95-6038, UA2715^b^	Ancona, Italy	Clinical	1995	HIV+	CSF	VNII/VNIV	aADα	ND	ND	ND	[35, 38]
WM 2617	MC-420 (a)	India, Tamil Nadu, Velore	Clinical	1995	HIV+	CSF	VNI/VGI	αABa	VNI/VGII	2.384	0.8	[19]
WM 05.272	H0058-I-1959	Colombia, Cúcuta	Clinical	2003	HIV-	CSF	VNI/VGII	αABa	VNI/VGII	2.466	0.72	[19]
WM 05.459	LMM 558	Brazil, Piaui, Castelo do Piaui	Clinical	1996	HIV-	CSF	VNI/VGII	αABa	VNI/VGII	2.634	0.75	[19]
WM 05.532	LMM 868	Brazil, Maranhão, Caxias	Clinical	2001	HIV-	CSF	VNI/VGII	αABa	VNI/VGII	2.634	0.8	[19]
WM 628^d^	CBS10080	Melbourne, Australia	Clinical	1988	HIV+	CSF	VNIII	αADa	ND	ND	0.73	[62]
WM 1355^d^	TBS 54	Madras, India	Clinical	ND	HIV+	ND	VNIII	αADa	VNIII	2.218	ND	This study
WM 1529^d^	RKI-M364/98	Germany	Clinical	1998	HIV-	CSF	VNIII	αADa	ND	ND	ND	[63]
WM 01.80^d^	KW5a	Kuwait	Clinical	1996	ND	CSF	VNIII	αADa	VNIII	2.242	ND	This study
WM 01.123^d^	CBS132; ATCC 32045	Italy	Environmental	1978	NA	Fermenting fruit juice	VNIII	αADa	VNIII	2.101	0.69	[42]
WM 846	H99, CBS 8710, ATCC 208821	USA	Clinical	1978	HIV-	Hodgkins	VNI	Aα	ND	ND	0.56	[64]
WM 01126	JEC20	USA	NA	ND	NA	NA	VNIV	Dα	ND	ND	0.83	[41]
WM 02.35	CDC R265	Canada	Clinical	2001	HIV-	CSF	VGIIa	Bα	ND	ND	0.71	[43]
WM 02.38	CDC R272	Canada	Clinical	2001	HIV-	BAL	VGIIb	Bα	ND	ND	0.6	[44]

Note:

^*a*^ In 2001, this isolate was reported as an AFLP genotype 1 (corresponding to VNI), serotype A veterinary isolate from a cat in Australia [61]. In 2008, the same isolate was characterized as AFLP genotype 1B (corresponding to VNII), serotype A, mating type α diploid strain [27].

^*b*^ These isolates were previously genotyped as VNIII with the M13 primer [38].

^c^ 2.300–3.000 = highly probable species identification, 2.000–2.299 = secure genus identification with probable species identification (Bruker, Daltonics)

^d^ These VNIII strains were used in different analyses as a comparison to VNII/VNIV hybrid isolates.

ND: No data

NA: Not applicable.

### Intra-varietal (VNI/VNII, αAAα) hybrids

**Fig 1:**
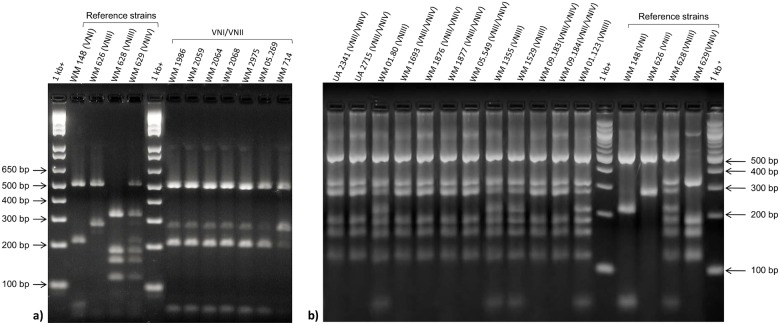
PCR-RFLP profiles of the a) VNI/VNII b) VNII/VNIV hybrid isolates as well as *C*. *neoformans* molecular types reference strains obtained via double digest of the *URA*5 gene with *Hha*I and *Sau*96I. 1-kb^+^ DNA ladder (Invitrogen, Carlsbad, USA) was used as molecular size marker. VNIII isolates were used for comparison to VNII/VNIV hybrids.

**Fig 2:**
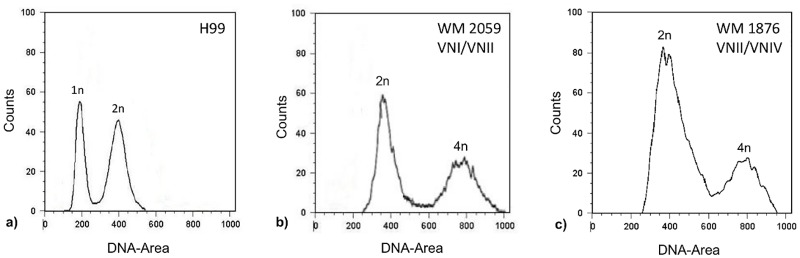
Flow cytometry profiles of a) haploid control strain (H99, VNI), b) an example of αAAα (WM 2059, VNI/VNII) and c) aADα hybrid (WM 1876 VNII/VNIV) isolates after staining with the fluorescent dye propidium iodide. 1n, 2n, and 4n indicate nuclear content. The *x*-axis indicates fluorescence intensity reflecting the DNA content, and the *y*-axis indicates cell counts.

Comparison of the *URA5*-RFLP profiles of all studied isolates with those of the standard reference strains revealed a unique hybrid pattern consistent with a composite of molecular types VNI and VNII for seven serotype A isolates (Fig 1a). In 2001, one of the herein identified VNI/VNII hybrids, WM 714, was reported as AFLP genotype 1 (corresponding to VNI), serotype A isolate [61]. In 2008, the same isolate was characterized as AFLP genotype 1B (corresponding to VNII), serotype A, mating type α diploid strain [27]. Determination of the ploidy using flow cytometry analysis showed that all potential hybrid isolates contained ~2× more DNA (Fig 2b) than the haploid control strain (Fig 2a), confirming that they were diploids. Fluorescent microscopy showed that all cells were uninucleate. Using Luminex xMAP technology, the *C*. *neoformans* specific probe (CNN b), *C*. *neoformans* var. *grubii* probe (CNN 1b), VNI specific probe (CNN 1a/1b) and VNII specific probe (CNN 1c) gave positive signals for all VNI/VNII hybrid isolates (data not shown). Therefore, these isolates were identified as hybrids of the molecular types VNI and VNII. Restriction enzyme analysis of the *CAP59* gene with *Bsm*FI and *Hpa*II showed that all VNI/VNII hybrids had only the serotype A allele (S1 Fig). Mating type and serotype- and mating-type-specific PCR analysis results for the VNI/VNII hybrids in comparison with the reference strains are shown in S3 Table, indicating that all the VNI/VNII hybrids were αAAα (Table 1). Analysis of the VNI/VNII hybrid isolates revealed many heterozygous sites when both mating type (*SXI1α*, *STE20α*) and other genomic loci (*GPD1*, *PLB1*, *SOD1*, *URA5* and IGS1) were sequenced, indicating that these α/α hybrids were most likely derived from same-sex mating through fusion between two genetically distinct α cells of the same serotype.

### Inter-varietal (VNII/VNIV, aADα) hybrids

**Fig 3:**
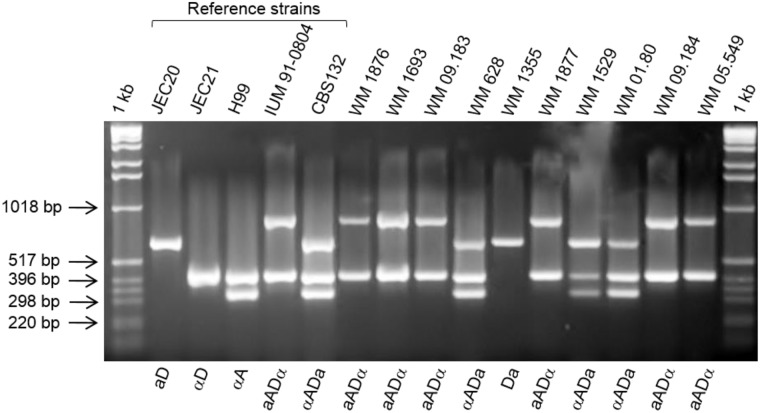
Results of mating-serotype multiplex PCR for the hybrid isolates and reference strains. 1-kb = DNA ladder (Invitrogen, Carlsbad, USA).

Comparison of the *URA5*-RFLP profiles of all studied isolates with those of the standard reference strains revealed a novel mixed pattern between the molecular types VNII and VNIV for six clinical isolates and two environmental isolates. The profiles of the VNII/VNIV hybrids lack the VNI specific bands characteristic for the VNIII hybrids, which are a combination of VNI, VNII and VNIV (Fig 1c). Using Flow cytometry, these hybrid strains were shown to contain ~2× more DNA to that found in the reference haploid strain (Fig 2c). Fluorescent microscopy showed that all cells were uninucleate. Restriction enzyme analysis of the *CAP59* gene with *Bsm*FI and *Hpa*II showed the presence of both serotype A and D alleles for the VNIII hybrids, except for the strain CBS132 (WM 01.123), which showed only the serotype D allele (S2a Fig), as no digestion product cuts were obtained with *Hpa*II. Moreover, the *Hpa*II enzyme could not digest the PCR product of VNII/VNIV hybrids (S2b Fig), which is most likely due to the fact that the *CAP59* primers used in this RFLP analysis are not able to amplify the Dα allele. In the mating type analysis, all VNIII hybrids yielded amplicons with the primer sets specific for *MFα* (MFαF/MFαR) and *MF****a*** (MFaF/MFaR). Positive amplifications were obtained for the VNII/VNIV hybrids for both the *STE12α* and *STE12****a*** loci. Interestingly, simultaneous mating-serotype multiplex PCR showed the **a**ADα sero/mating type combination for all VNII/VNIV hybrids and the αAD**a** sero/mating type combination for all VNIII strains (Table 1), except for strain WM 1355, which showed a D**a** pattern (Fig 3). The sero/mating type of the Italian AD hybrids (UA2341 and UA2715) had been previously assigned as **a**ADα by allele-specific PCR of the mating type locus genes and sequencing of the *MAT* gene [38]. Analysis of the allelic patterns of the selected genes for the AD hybrids showed that only one isolate, WM 01.80, with αAD**a** sero/mating type pattern, was heterozygous at all loci. All other hybrids were heterozygous in more than 79% of the studied loci, except for the strain CBS132, which showed only 50% of heterozygosity (Table 2). For the isolates UA2341 and UA2715, the percentage of heterozygosity was previously reported as 80% and 87%, respectively [38].

**Table 2:** Characteristics of AD hybrid isolates and reference strains investigated in this study and allelic patterns and percentage of heterozygosis of the genes investigated.

Strain	molecular type	Mating/ serotype	*IGS1*	*SOD1*	*LAC1*	*PLB1*	*CAP59*	*GPD1*	*CAP10*	*URE1*	*GPA1*	*CNA1*	*PAK1*	*LBP*	*MDT*	*CDK*	% HZ
WM 1876	VNII/VNIV	**a**ADα	AD	AD	AD	A	AD	A	AD	AD	AD	D	AD	AD	AD	AD	79
WM 1693	VNII/VNIV	**a**ADα	AD	AD	AD	AD	A	D	AD	AD	AD	AD	AD	AD	D	AD	79
WM 09.183	VNII/VNIV	**a**ADα	AD	AD	AD	AD	AD	A	AD	AD	AD	AD	AD	AD	AD	AD	93
WM 1877	VNII/VNIV	**a**ADα	AD	AD	AD	AD	AD	A	AD	AD	AD	AD	AD	AD	AD	AD	93
WM 09.184	VNII/VNIV	**a**ADα	AD	AD	AD	AD	AD	A	AD	AD	AD	AD	AD	AD	AD	AD	93
WM 05.549	VNII/VNIV	**a**ADα	AD	AD	AD	AD	AD	A	AD	AD	AD	AD	AD	D	D	AD	79
UA2341	VNII/VNIV	**a**ADα	AD	AD	AD	AD	A	A	AD	AD	AD	AD	AD	AD	D	AD	79
UA2715	VNII/VNIV	**a**ADα	AD	AD	AD	AD	AD	A	AD	AD	AD	D	AD	AD	AD	AD	86
WM 628	VNIII	αAD**a**	AD	AD	AD	AD	AD	AD	AD	AD	A	AD	AD	AD	AD	AD	93
WM 1529	VNIII	αAD**a**	AD	AD	AD	AD	AD	AD	AD	AD	AD	D	AD	A	AD	AD	86
WM 01.80	VNIII	αAD**a**	AD	AD	AD	AD	AD	AD	AD	AD	AD	AD	AD	AD	AD	AD	100
WM 1355	VNIII	αAD**a**	AD	AD	AD	AD	AD	AD	AD	AD	A	D	AD	A	AD	AD	79
CBS132	VNIII	αAD**a**	AD	AD	D	A	D	AD	AD	D	A	D	AD	AD	AD	D	50
H99	VNI	αA	A	A	A	A	A	A	A	A	A	A	A	A	A	A	0
JEC21	VNIV	αD	D	D	D	D	D	D	D	D	D	D	D	D	D	D	0

HZ: Heterozygosis.

### Application of MALDI-TOF MS for the identification of hybrid isolates

According to the MALDI-TOF MS manufacturer’s criteria (spectral score ≥2.0), all 20 studied hybrid isolates were identified correctly to the species level (100% concordance with the reference spectra of five *C*. *neoformans* and two *C*. *gattii* strains currently included in the MALDI-TOF Biotyper BDAL MSP library (Bruker Daltonics). Run against our in-house spectral database for the *C*. *neoformans/C*. *gattii* species complex, containing spectra for 160 strains (20 per major molecular type), all hybrid isolates except two, were identified as hybrids belonging to the expected group (identification scores of 2.101 to 2.634) (Table 1). One VNI/VNII isolate (WM 2975) was recognized as a VNIII isolate. The VNI/VGI isolate (WM 2617) was recognized as a VNI/VGII isolate, likely because there is only one VNI/VGI hybrid in the database and the closest match was a VNI/VGII hybrid. The results of the present study have added 120 novel spectra for the different hybrid genotypes.

### *G*. *mellonella* virulence studies

**Fig 4:**
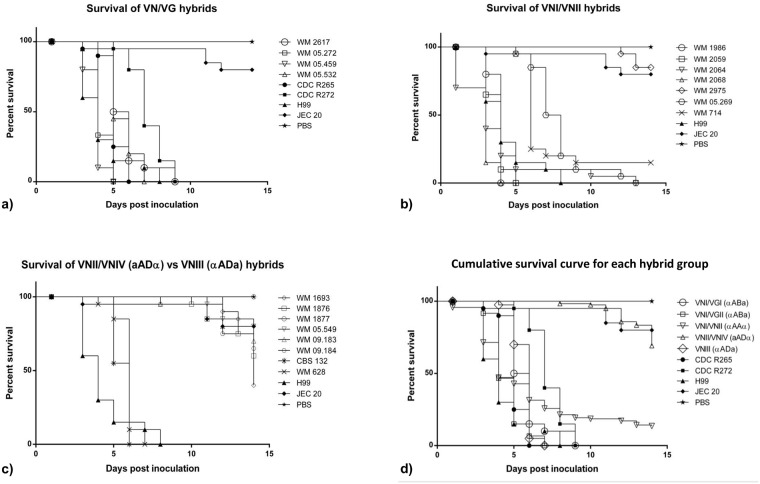
Survival curves of larvae infected with the different hybrid strains. a) VN/VG hybrids, b) VNI/VNII hybrids, c) VNII/VNIV versus VNIII hybrids, and d) cumulative survival curves comparing the different hybrid groups.

All hybrid isolates killed the *G*. *mellonella* larvae at 37°C, but the rate of killing varied between the hybrid group strains. All VN/VG hybrids caused 100% mortality within 9 days of infection (Fig 4a). All clinical VNI/VNII strains caused 100% mortality before day 14, except for strain WM 2975 (which might be due to technical problems during inoculation) and the veterinary isolate WM 714, which showed 20% and 85% mortality, respectively (Fig 4b). Clinical and environmental VNII/VNIV (**a**ADα) hybrids caused 0%-60% mortality, whereas the VNIII (αAD**a**) hybrids caused 100% mortality within 14 days (range 6–7 days) (Fig 4c). None of the larvae inoculated with PBS died within the study period.

Fig 4d shows cumulative survival curves of larvae infected with different hybrid groups. S4 Table, summarizes the cumulative survival of larvae for each hybrid group and the control strains. The VNI/VGII hybrid strains were significantly more virulent than the VNI/VGI strain (*p*<0.001). The highly virulent strain H99 (Aα) was significantly more pathogenic than the αAD**a** (VNIII) (*p*<0.001), the VNI/VGI (αAB**a**) (*p* = 0.002), the VNII/VNIV (**a**ADα) (*p*<0.001) and the VNI/VNII (αAAα) (*p* = 0.016) hybrids. There was no significant difference in survival rates in larvae infected with H99 and the VNI/VGII hybrids (αAB**a**) (*p* = 0.547) and the high virulent Vancouver Island outbreak strain, CDC R265 (VGIIa, Bα) (*p* = 0.070). All hybrid strains were more virulent than the reference strain JEC20 (Da) (*p*<0.001) except for the VNII/VNIV hybrids (*p* = 0.457) and strain WM 2975 (VNI/VNII) (*p* = 0.882). All the VN/VG hybrids were also more virulent than the less virulent Vancouver Island outbreak strain, CDC R272. A clear rank order of pathogenicity of hybrid groups and control strains was found with H99 (Aα), CDC R265 (Bα), VNI/VGII (αAB**a**) > VNI/VGI (αAB**a**), VNIII (αAD**a**) > CDC R272 (Bα) > VNII/VNIV(**a**ADα), JEC20 (D**a**). Larvae infected with the VNI/VNII hybrids exhibited 50% mortality by day 4 post-infection. However, as the larvae infected with the strains WM 2975 and WM 714 exhibited a lower killing rate, based on statistical analysis this group of hybrids showed only a significant difference from the VNI/VGII hybrids (*p* = 0.005), H99 (*p* = 0.016), VNII/VNIV hybrids (*p*<0.001) and JEC20 (*p*<0.001) and therefore could not be included in the pathogenicity rank order.

### Cell and capsule size

**Fig 5:**
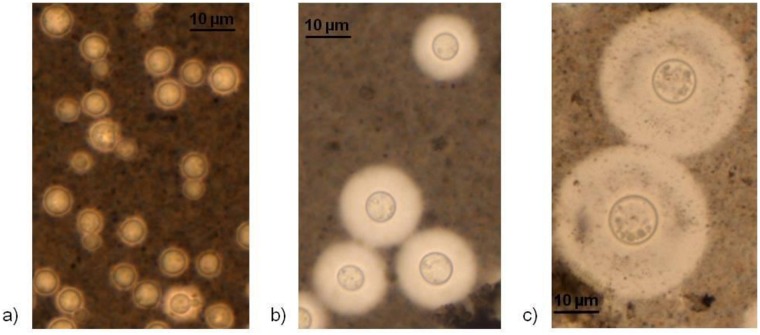
Estimation of total cell and capsule size by India ink staining. a) Cells grown overnight in YPD broth. b) Cells recovered from larvae infected with a hybrid isolate (10^6^ cells/larva) c) giant cells recovered from larvae infected with hybrid isolate, WM 1986 (10^6^ cells/larva).

**Fig 6:**
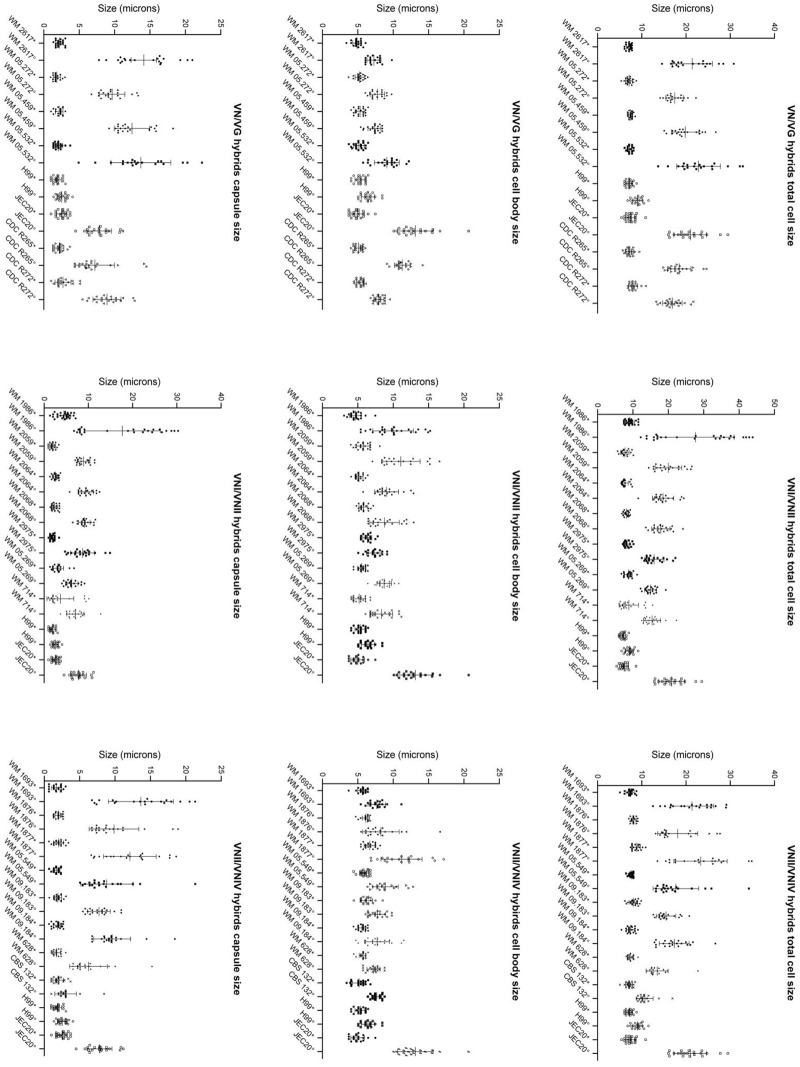
Distribution of total cell size, cell body size and capsule diameter of individual isolates in each hybrid group and the haploid control strains after overnight growth in YPD broth (*) and recovered from the *G*. *mellonella* larvae after inoculation of 10^6^ yeast cells (°). Bars denote the average of distribution and standard deviation.

The total size of hybrid cells (including capsule) following overnight growth in YPD broth ranged between 5.08–15.58 μm (mean value 7.91 μm) (Fig 5a) whereas those recovered from larvae ranged between 8.81–43.86 μm (mean 18.54 μm) (Fig 5b), which was more than 2-fold greater than the average size observed *in vitro* (7.9 μm). This was due to both an increase in the cell body and capsule size (Fig 6). The total size of haploid cells (including capsule) following overnight growth in YPD broth ranged between 5.44–10.89 μm (mean value 7.91 μm) whereas those recovered from larvae ranged between 6.81–29.48 μm (mean 16.38 μm). Therefore, the mean value of the total cell size of the hybrids was bigger than the haploid control strains before and after injection to larvae. Among all hybrid groups and control strains, the VNI/VGI hybrid showed the biggest average total cell and capsule size during infection in the *G*. *mellonella* larvae and strain H99 showed the smallest of all cell sizes (S3 Fig). Comparing the VNIII and the VNII/VNIV hybrids, the VNII/VNIV hybrids showed a notably bigger average capsule and total cell size during infection in the *G*. *mellonella* larvae and the VNIII strains showed the smallest capsule and total cell size compared to the other hybrid groups (S3 Fig).

There was a significant difference in the ratio of capsule size to the total cell size of cells recovered from YPD broth between different groups of hybrids and control strains (*p*<0.001). Therefore, the mean change in the ratio of the capsule size to the total cell body size of the cells before and after injection in the larvae for all hybrid groups and control strains was calculated with 95% confidence intervals (Table 3). The results showed a significant difference for all hybrid groups (*p*≤0.001). As per colour code in Table 3, VNI/VGI, VNI/VGII and VNII/VNIV hybrids were comparable and showed the biggest change compared with the other hybrids and control strains. The VNI/VNII hybrids and the strain CDC R272 were comparable and the VNIII hybrids did not group with any other hybrid group or the control strain. The strains CDC R265, JEC20 and H99 were comparable with the smallest change in ratio.

**Table 3:** Changes in the mean ratio of capsule size to the total cell body size of cells before and after injection to the larvae with 95% confidence intervals and geometric mean of capsule size and mean ratio of capsule:total cell size for different hybrid groups and haploid reference strains.

Hybrid group/ control strain	Change in mean ratio pre-post injection	95% Confidence Interval for change in mean ratio		*p*-value for change in mean ratio	Geometric mean of capsule size	95% confidence interval for geometric mean		Mean ratio capsule:total cell size	95% confidence interval for mean	
		Lower bound	Upper bound			Lower bound	Upper bound		Lower bound	Upper bound
VNI/VGI	0.34	0.30	0.38	<0.001	5.42	5.00	5.87	0.52	0.50	0.54
VNI/VGII	0.31	0.29	0.34	<0.001	8.21	7.68	8.77	0.61	0.59	0.63
VNII/VNIV	0.30	0.28	0.32	<0.001	6.24	6.02	6.46	0.50	0.49	0.51
CDC R272	0.19	0.14	0.25	<0.001	5.67	5.00	6.42	0.47	0.44	0.50
VNI/VNII	0.17	0.14	0.19	<0.001	6.33	6.00	6.68	0.51	0.49	0.52
VNIII	0.11	0.06	0.16	<0.001	2.82	2.64	3.01	0.31	0.29	0.32
CDC R265	0.08	0.03	0.13	0.001	4.22	3.98	4.46	0.39	0.37	0.40
JEC20	0.04	-0.01	0.08	0.084	6.03	5.63	6.45	0.48	0.46	0.50
H99	0.02	-0.02	0.07	0.276	2.35	2.18	2.55	0.26	0.24	0.27

In addition to capsule enlargement, giant cells (above 30 μm) were observed in all three hybrid groups, which was due to both capsule enlargement and increase of the cell body size (Fig 5c). The proportion of giant cells recovered from the larvae infected with strains WM 05.532 (VNI/VGII) and WM 1877 (VNII/VNIV) was 10%, for strain WM 2617 (VNI/VGI) and WM 05.549 (VNII/VNIV) it was 5%, and for strain WM 1986 (VNI/VNII) it was 4.8%. There was no correlation between median survival time with mean change in ratio of capsule size to the total cell body size of cells of each hybrid group or the control strains before and after injection to the larvae (correlation coefficient: -0.009; *p* = 0.983), median ratio of capsule size to the total cell body size of cells before (correlation coefficient: 0.051; *p* = 0.896) and after (correlation coefficient: 0.051; *p* = 0.896) injection to the larvae, median total cell size before (correlation coefficient: 0.306; *p* = 0.424) and after (correlation coefficient: 0.185; *p* = 0.634) injection to the larvae, median capsule size before (correlation coefficient: 0.185; *p* = 0.633) and after (correlation coefficient: 0.077; *p* = 0.844) injection to the larvae. Also, no correlation was found between median survival time with median total cell size (correlation coefficient: -0.141; *p* = 0.52), median cell body size (correlation coefficient: -0.02; *p* = 0.929), median capsule size (correlation coefficient: -0.163; *p* = 0.459) and median ratio of capsule size to the total cell size (correlation coefficient: -0.083; *p* = 0.706) after recovering the cells from infected larvae for each individual hybrid or the control isolates. Moreover, there was no correlation between the median survival time and proportion of giant cells (correlation coefficient: 0.022; *p* = 0.922).

### Virulence factors

**Fig 7:**
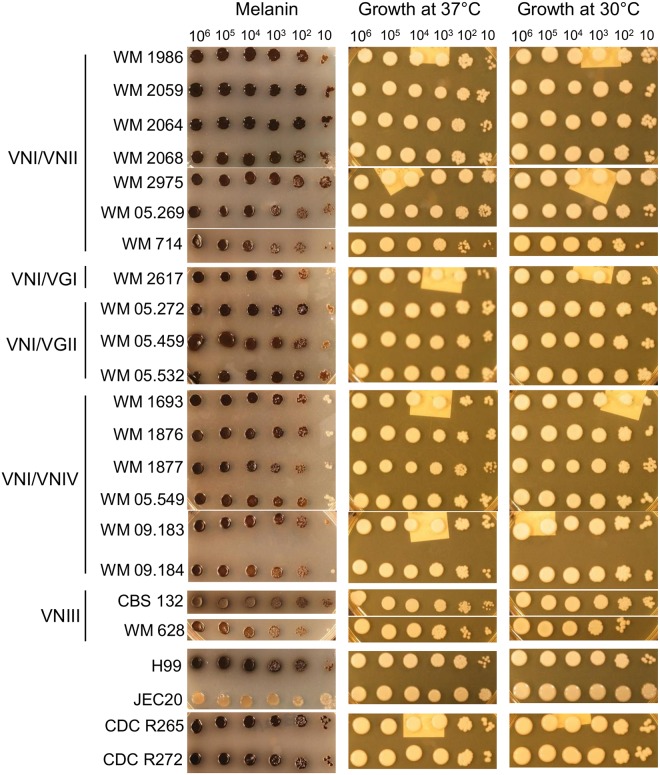
*In vitro* assays of melanin production on L-DOPA agar, growth at 37°C on YPD agar and growth on YPD agar at 30°C as growth control for hybrid and haploid strains.

All hybrids and the haploid reference strains grew well at 37°C, similar to their growth at 30°C, although their rate of growth varied slightly (Fig 7). The production of melanin during growth on L-DOPA medium at 37°C was observed in all hybrids and the haploid reference strains (H99, CDC R265, CDC R272) but not in the low virulent JEC20 (D**a**) strain (negative control) (Fig 7). Based on light microscopy all tested strains produced a capsule after incubation at 35°C in RPMI-1640 agar medium, as demonstrated by negative staining with India ink (Fig 5). All isolates secreted phospholipase as indicated by white zones of precipitation around the colonies. Zone sizes varied depending on the isolate. Phospholipase production, expressed as the Pz value, of tested isolates is summarized in Table 1. Means of phospholipase zone (Pz) index were 0.8 (±0) for VNI/VGI, 0.76 (±0.04) for VNI/VGII, 0.65 (±0.08) for VNI/VNII, 0.61 (±0.06) for VNII/VNIV and 0.71 (±0.02) for VNIII.

### Antifungal susceptibility

All hybrid isolates were susceptible to all tested drugs except for 5-flucytosine, which was intermediate (MIC = 8) for WM 05.272 (VNI/VGII, αAB**a**) when compared with the breakpoints for antifungal susceptibility established for *Candida* spp.

**Fig 8:**
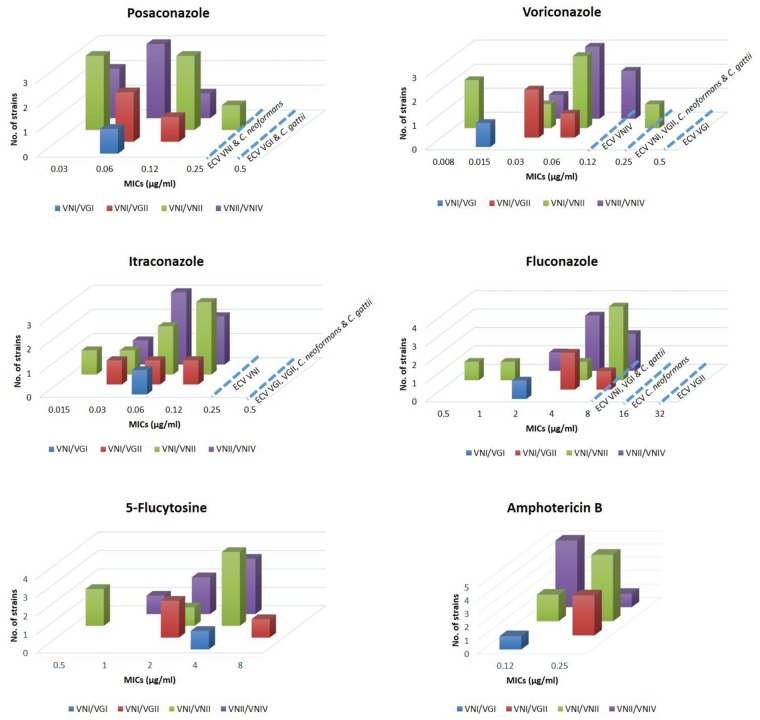
MICs (μg/ml) of azoles, 5-flucytosine and amphotericin B against *Cryptococcus* hybrid strains compared with epidemiological cut off values (ECVs) for *Cryptococcus neoformans*/*Cryptococcus gattii* species complex.

No significant differences were found in the MICs between VNI/VNII, VNII/VNIV and VN/VG hybrid groups (*p* = 0.579). MICs were significantly different for each tested drug (*p*<0.001), except for posaconazole and itraconzole, which showed similar activity (*p* = 0.973). Fig 8 shows azoles, 5-flucytosine and amphotericin B MIC (μg/ml) distributions for *Cryptococcus* hybrid strains. Azoles were compared with epidemiological cut off values (ECVs) for *Cryptococcus neoformans*/*Cryptococcus gattii* species complex described previously [65]. The MICs of all hybrids against the azoles were below or equal to the parental molecular types and species ECVs (Fig 8). Since no ECVs are reported for 5-flucytosine and amphotericin B, geometric mean of all hybrid genotype groups were compared with the calculated GM of published relevant molecular types, serotypes and species (Table 4). Previously published data are listed in S5 Table. The geometric mean of the VNI/VNII and VNII/VNIV hybrids was lower than the published GMs against the parental molecular types, serotypes and species for amphotericin B and 5-flucytosine. Amphotericin B MIC for the VNI/VGI hybrid was lower than published MICs of all relevant molecular types, serotypes and species. However, 5-flucytosine MIC for the VNI/VGI hybrid was higher than published MICs of all parental molecular types, serotypes and species. The GM of the VNI/VGII hybrids was equal or lower than the published GMs for VNI, VGII, serotypes A and B, *C*. *neoformans* and *C*. *gattii* for amphotericin B except for the serotype B for which the MICs GM was slightly higher. The GM of the VNI/VGII hybrids was lower than the published GMs for VNI, VGII, *C*. *neoformans* and *C*. *gattii* but higher than serotypes A and B for 5-flucytosine (Table 4).

**Table 4:** MICs (μg/ml) geometric mean (GM) values of 5-flucytosine and Amphotericin B against different cryptococcal hybrid groups and calculated combined overall previously published MICs ranges and GM of each haploid group within the *C*. *neoformans/C*. *gattii* species complex.

Hybrid group	VNI/VNII (αAAα)	VNII/VNIV (aADα)	VN/VG (αABa)	VNI	Serotype A	Serotype D	Serotype AD	*C*. *neoformans*	VGI	VGII	Serotype B	*C*. *gattii*
	GM	GM	GM	GM	GM	GM	GM	GM	GM	GM	GM	GM
**5-flucytosine**	2	2.519	3.363	3.43	3.123	3.204	4.443	3.6	1.667	4	1.66	3.304
**Amphotericin B**	0.205	0.14	0.21	0.56	0.25	0.156	0.174	0.335	0.356	0.44	0.239	0.363

## Discussion

Although most naturally-occurring *Cryptococcus* hybrid strains are clinical and environmental inter-varietal AD hybrids [8, 11, 12, 23, 32, 34, 61, 66], several clinical inter-species hybrids between serotypes A and B [18, 19] and B and D [17] have been described. Additionally, naturally-occurring clinical and environmental intra-varietal αAAα hybrids have been reported [26].

In this study, seven natural αAAα hybrid strains from clinical and veterinary sources were identified and characterized. These strains were homozygous for the serotype A-specific allele and the *MAT*α mating type locus. This result is consistent with the α-mating type predominance in the serotype A population [26]. The herein reported hybrids between VNI and VNII are either additional proof for previously reported intra-varietal hybrids between VNII and VNB [26, 27] or in fact represent a new hyrbid type, for which final confirmation is subject to further studies, which may need to include the originally described VNII/VNB hybrid strains [26]. Our findings, taken together with previous reports of α/α diploids resulting from same-sex mating, emphasizes the importance of this process for generating genetic diversity and to assist this fungus to compensate for a mainly unisexual population structure. Our data are also consistent with the notion that intra-varietal hybridization is likely to occur at a low frequency in nature [26]. These hybrids may not be easily recognized due to sensitivity limitations associated with most of the commonly used typing techniques. The detection of αAAα hybrids in both Colombia and Australia as described in this study, suggests that the intra-varietal same-sex mating process is not restricted to specific geographic regions or less likely that the hybrid strains clonally expanded and became widely dispersed.

The new pattern VNII/VNIV is associated to mating type **a**ADα hybrids, whereas the pattern VNIII is associated to αAD**a** allelic pattern. These findings suggest that, in the **a**ADα hybrids here investigated, the *URA5* allele A is from the VNII strains and therefore that A**a** *MAT* locus originated from the VNII population. This is also supported by the existence of natural isolates belonging to molecular type VNII and mating type α as shown in the case of strain IUM 96–2828 [40]. The results obtained by M13 PCR-fingerprinting showed different profiles for αAD**a** and **a**ADα isolates indicating genetic variation between them. This was consistent with the previously reported AFLP genotypes of αAD**a** and **a**ADα strains [66].

The allelic pattern obtained for strain CBS132 was identical to the one reported previously [38], confirming the reproducibility of the method and the stability of the hybrid genome. The fact that the majority of AD strains are heterozygous for many but not all genetic markers analysed might be due to gene conversion, deletion and/or chromosome loss. Loss of heterozygosity is likely to be common following hybridization between strains of serotype A and D. Allelic losses for many of the loci examined in the AD strains have been previously reported [10, 38, 67]. Genetic and phenotypic changes have been reported for *C*. *neoformans* strains during prolonged asexual propagation and suboptimal storage in the laboratory [7, 68]. It has been suggested that hybridization followed by differential loss of heterozygosity could be an important mechanism for generating genetic variation in C. *neoformans* [12].

The current study investigated the genome heterozygosis of several AD hybrids, found no correlation between molecular type or mating type allelic pattern and level of heterozygosity in the hybrid isolates. A high level of heterozygosity was obtained for the majority of AD hybrids studied, suggesting that heterozygosis could enhance the fitness of *C*. *neoformans*. A variable percentage of heterozygosis was observed in the AD hybrids even within each sero/mating type group. The fact that some loci were homozygous and that heterozygous regions were distributed randomly supports the hypothesis that *C*. *neoformans* hybrids originated from a post-meiotic event [33].

MALDI-TOF MS as shown previously can not only distinguish between *C*. *neoformans* and *C*. *gattii* [69], but also between *C*. *neoformans* var. *neoformans* from *C*. *neoformans* var. *grubii* and AD hybrids [70]. Furthermore, it can recognise different cryptococcal genotypes [70]. The current study showed that MALDI-TOF can also identify cryptococcal hybrids. Moreover, it strongly suggests that MALDI-TOF can distinguish inter-species, intra-varietal and inter-varietal hybrids and identify their composite molecular types in agreement with the DNA-based typing methods. Finally it confirms the highly discriminatory potential of MALDI-TOF MS as a powerful technique for the routine identification of hybrids from other strains and from each other within the *C*. *neoformans*/*C*. *gattii* species complex. The discordant result in identification of one VNI/VNII isolate, which was recognized by MALDI-TOF MS as a VNIII isolate, and one VNI/VGI isolate, which was recognized as a VNI/VGII isolate, is most likely due to the lack of reference spectra for hybrid strains in the database. Future improvement of the database by addition of more spectra obtained form hybrids should reduce these misidentifications.

To our knowledge this is the first study in which the pathogenicity of different groups of hybrids was compared in a *G*. *mellonella* model. *G*. *mellonella* is a recent model applied to study *Cryptococcus* spp. pathogenicity [71]. It takes advantage of the phagocytic haemocytes which phagocyte and kill bacterial and fungal cells by a mechanism similar to that used by human neutrophils, namely, via the production of superoxide [72]. *G*. *mellonella* larvae have been used to study the pathogenesis of other fungal infections which also affect humans, such as those caused by *Candida albicans*, *Aspergillus fumigatus* and *A*. *flavus* [73–76]. The results showed that hybrid strains containing the Aα mating type locus are highly virulent in the *G*. *mellonella* model. These results are consistent with a previous study by Kwon-Chung *et al*., which reported that there is a relationship between mating type and virulence, with *MAT*α being more virulent than *MAT***a** strains [41]. The VNI/VGII (αAB**a**) hybrids were found to be as virulent as the haploid virulent strains H99 and CDC R265. The VNI/VGI (αAB**a**) and the VNIII (αAD**a**) hybrids were virulent but less so than strain H99. Similarly, it has been reported that hybrid strains containing the Aα mating type locus were highly virulent in the murine tail vein injection model and haploid Aα strains were more virulent than haploid Aa and D**a** strains [35]. Haploid and diploid strains containing the Dα allele have exhibited a wide range of virulence [35]. The virulence of AD hybrids has been reported intermediate between the reference strains H99 (Aα) and JEC20 (D**a**), with the **a**ADα hybrids showing much lower virulence [35]. Two other studies have shown that haploid Aa strains are considerably less virulent than strain H99 [77, 78]. Virulence of eight αAD**a** hybrid strains in an experimental intravenous mouse model was similar to strain H99 [34]. Lengeler *et al*. tested two αAD**a** and two **a**ADα hybrid strains and found that one αAD**a** strain was virulent but less than strain H99. The rest of the tested strains were moderately virulent [10]. Another study on laboratory-generated αAD**a** and **a**ADα hybrid strains suggested that their virulence is close to the haploid serotype A parental strains [37]. However, our results showed that αAD**a** hybrids are virulent, but less so than strain H99. The **a**ADα hybrid strains were not as virulent and grouped together with the low avirulent serotype D strain JEC20. The difference in virulence between αAD**a** and **a**ADα strains is likely due to the difference in genotype. This suggests that the serotype origin of the mating type plays a role in virulence and the presence of Aα confers an advantage in virulence as it has been suggested in previous studies [34, 35] with the α allele from serotype A being associated with higher virulence in AD hybrids [35]. This hypothesis is supported by the previous observation of reduced pathogenicity of **a**ADa hybrids compared with other AD hybrids [37]. The difference between virulence of natural and laboratory generated hybrids might be due to the independent origin of the natural isolates, with different genetic backgrounds and a considerable mitotic expansion in nature [12].

The results of this study showed that natural αAAα hybrids are as virulent as or less than Aα (H99) haploid strains, which confirmed previous findings showing that isogenic αAAα hybrid strains are slightly less virulent than haploid serotype A strains, but still highly virulent, suggesting that higher ploidy may modestly reduce virulence in the host [37].

It has been shown that *C*. *neoformans* undergoes a large increase in both capsule size and cell body size during mammalian pulmonary infection [79, 80] and in the *G*. *mellonella* infection [81]. Similar changes occurred in the hybrid strains, as they manifested a significant increase in the average capsule and total cell size during *G*. *mellonella* larvae infection. In addition to capsule enlargement, the appearance of giant cells has been reported in cryptococcal infections in the murine [80, 82] and *G*. *mellonella* [81] models, as was also shown in the current study for cryptococcal hybrid strains. Emergence of giant cells in both the mouse and the *G*. *mellonella* models indicates that this phenomenon is not host specific. Although giant cells, which have been recovered from *G*. *mellonella* had similar properties to the giant cells obtained from mice, the ones isolated from mice were larger [80]. A possible explanation for this is the length of infection, which is longer in mice and gives more time to yeast cells to become larger than in the insect model due to short lifespan of the infected larvae [81], reemphasising that the formation of giant cell is an age-dependent phenomenon [80]. This may also explain the observations of the current study, which showed a bigger capsule and total cell size for the VNII/VNIV hybrids compared with the VNIII hybrids, or the serotype D strain JEC20 compared with the high virulent serotype A strain H99, or the VNI/VGI hybrids compared with the VNI/VGII hybrid, with the later having a lower median survival time, as the cells were recovered from the larvae after they died.

Except for the low virulent Vancouver Island outbreak haploid strain CDC R272, other studied haploid strains had the least mean change in ratio of capsule to the total cell size pre versus post injection of the larvae compared with the hybrid isolate. This indicates that the capsule of the hybrid strains became bigger than the haploid strains during infection (Table 3). The mean ratio of capsule:total cell size was almost similar under *in vitro* and *in vivo* capsule induction conditions. The exception was the strain CDC R272, which showed a bigger mean ratio of capsule:total cell size *in vivo* compared to *in vitro* capsule induction conditions.

In terms of capsule production, the haploid reference strains had smaller ratios of capsule:total cell size than the hybrids. The results of this study show no correlation between capsule/total cell size and virulence. This was consistent with a previous study on virulence of giant cells, for which the same virulence capacity as regular size cells had been observed [81]. Looking at the capsule size, no correlation between the *in vitro* capsule size and virulence or the capsule size indexes and allelic patterns of the *CAP59* and *CAP10* genes was found, confirming similar findings on AD hybrids reported previously [38].

*In vitro* virulence factor assays indicated that AD hybrids, except for strain CBS132, produced less melanin than the other hybrid groups. Within the VN/VG hybrids, the VNI/VGI showed weaker melanisation than the VNI/VGII hybrids. This may reflect their genotype differences and independent origins. All AD hybrids exhibited similar phenotypes regardless of the sero/mating type combination and their melanisation was intermediate compared to that of their respective haploid parents. No difference in melanin production was observed in other hybrid groups compared with their respective haploid parents. Studies on 19 AD hybrid isolates, found that the *LAC1* heterozygous pattern is not associated with differences in melanin production [38]. However, the current study showed that all AD hybrids are heterozygous at the *LAC1* locus, except for CBS132 (VNIII), which was homozygous for allele D. Interestingly, the intensity of melanin production in this strain was greater than that of the other AD hybrids, suggesting that heterozygosis at the *LAC1* locus may not only not enhance the production of melanin, it could even have an adverse effect on melanin production.

It has been reported that isolates of both *C*. *neoformans* and *C*. *gattii* produce similar amounts of phospholipase [83]. In addition, no statistically significant difference in phospholipase production was found in a study of phospholipase activity of 19 AD hybrid isolates [38]. This is consistent with our findings showing no significant difference between phospholipase activity between different hybrid groups and between hybrids and haploid controls regardless of the mating type and serotype combination. Moreover, clinical, veterinary and environmental hybrid isolates exhibited similar phospholipase production, confirming previous findings in clinical and environmental isolates of *C*. *neoformans* [83].

All hybrid strains behaved similarly in terms of their growth at human physiological temperature. The results of the current study showed no correlation between heterozygosity and growth at high temperature, but growth of the AD hybrids was slower than that of their haploid parents, regardless of being heterozygous or homozygous. Also, there was no correlation between virulence in the *G*. *mellonella* model and heterozygosity of the αAD**a** and **a**ADα hybrids. However, a previous study showed that heterozygous AD strains grew better than parental haploids at high temperatures and after UV irradiation, whereas homozygous hybrids grew more slowly than heterogygous hybrids and haploids at both high and low temperatures and after UV irradiation [38].

All tested antifungal compounds were active against all the tested hybrid strains, with fluconazole and 5-flucytosine having the highest MICs followed by amphotericin B. Posaconazole and itraconazole showed relatively low MICs compared with those of the other antifungal compounds. Voriconazole demonstrated excellent potency against each hybrid isolate. These results are comparable to the ones found for haploid *C*. *gattii* and *C*. *neoformans* strains [84–87]. Except for 5-flucytosine, posaconazole and itraconazole the VNI/VGII hybrids were less susceptible than the VNI/VGI hybrid isolate for all other tested drugs, reflecting previous finding, that the molecular type VGII is less susceptible than VGI [84, 88, 89]. Within the VNI/VNII group, WM 05.269 and WM 714 were more susceptible to 5-flucytosine, voriconazole, itraconazole and fluconazole compared with the other isolates in this group. Interestingly, in the virulence study these isolates produced less melanin and showed less virulence than the other isolates in the group. Overall, it can be concluded that hybridization may have a positive effect on antifungal susceptibility, as the studied hybrids showed equal or more susceptibility to antifungal agents compared to their parents.

In summary, hybrid strains comprise an important component of the *C*. *neoformans/C*. *gattii* species complex. They are recovered from clinical isolates and show relative similar virulent phenotypes in the experimental *G*. *mellonella* model, except for the VNII/VNIV (aADα) hybrids. Hybrids can undergo capsule enlargement and produce giant cells as their haploid parents, which might be a general adaptation tool for persistence and fungal survival in the host. This study has provided additional new insights in the overall population structure and the role of hybrids for the evolution of this important human/animal pathogen.

## Supporting Information

S1 Fig Restriction profiles of *CAP59* gene for VNI/VNII hybrid isolates and reference strains of different serotypes of *C*. *neoformans* on 3% agarose gel with *Bsm*FI and *Hpa*II restriction enzymes. Lane M, 1-kb^+^ DNA ladder (Invitrogen, Carlsbad, USA).
(PDF)

S2 Fig Restriction profiles of *CAP59* gene obtained with the *Bsm*FI and *Hpa*II restriction enzymes for VNIII hybrid isolates (a) and VNII/VNIV hybrid isolates (b) and reference strains separated on 3% agarose gel. 1kb^+^ = DNA ladder (Invitrogen, Carlsbad, USA).
(PDF)

S3 Fig Distribution of total cell size, cell body size and capsule diameter of all isolates in each hybrid groups and haploid control strains in Sabouraud (*) and recovered from the *G*. *mellonella* larvae after inoculation of 10^6^ yeast cells (°). Bars denote the average of distribution and standard deviation.
(PDF)

S1 Table PCR primer sequences used in this study. (PDF)

S2 Table Loci information outside the mating locus used for determination of α/α diploidization mechanism. (PDF)

S3 Table Mating type and serotype- and mating-type-specific PCR analysis results for the VNI/VNII hybrid isolates in comparison with reference strains. (PDF)

S4 Table Cumulative survival of larvae for each hybrid group and control strains. (PDF)

S5 Table Summary of previously published data of MICs (μg/ml) range and geometric mean (GM) values of 5-flucytosine and Amphotericin B agents against different cryptococcal strain groups. (PDF)
