# Supplementary figures and images for: Identification and Characterization of VNI/VNII and Novel VNII/VNIV Hybrids and Impact of Hybridization on Virulence and Antifungal Susceptibility Within the C. neoformans/C. gattii Species Complex
Source: PLoS One. 2016 Oct 20;11(10):e0163955. doi: 10.1371/journal.pone.0163955 (PMC5072701; doi:10.1371/journal.pone.0163955)

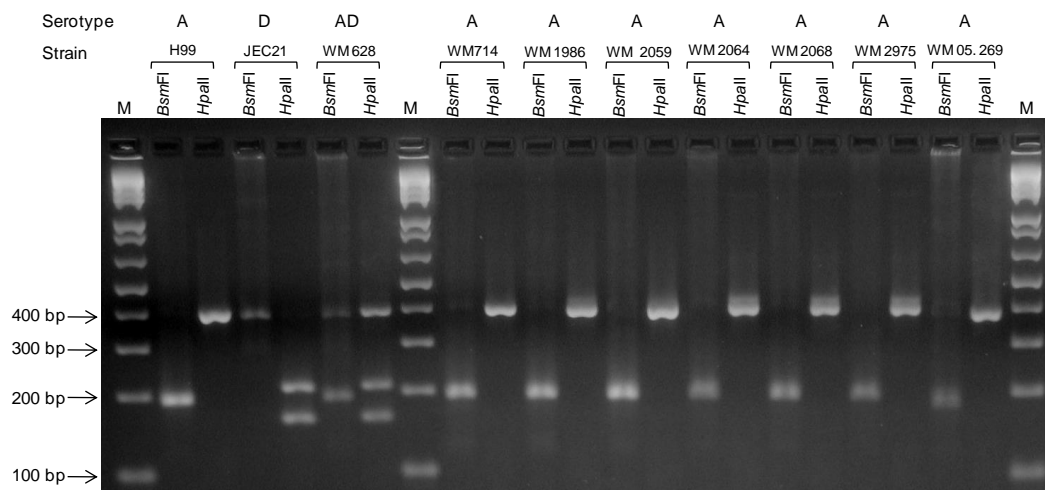

Supplement: S1 Fig — Lane M, 1-kb+ DNA ladder (Invitrogen, Carlsbad, USA). (PDF) [file pone.0163955.s001.pdf]

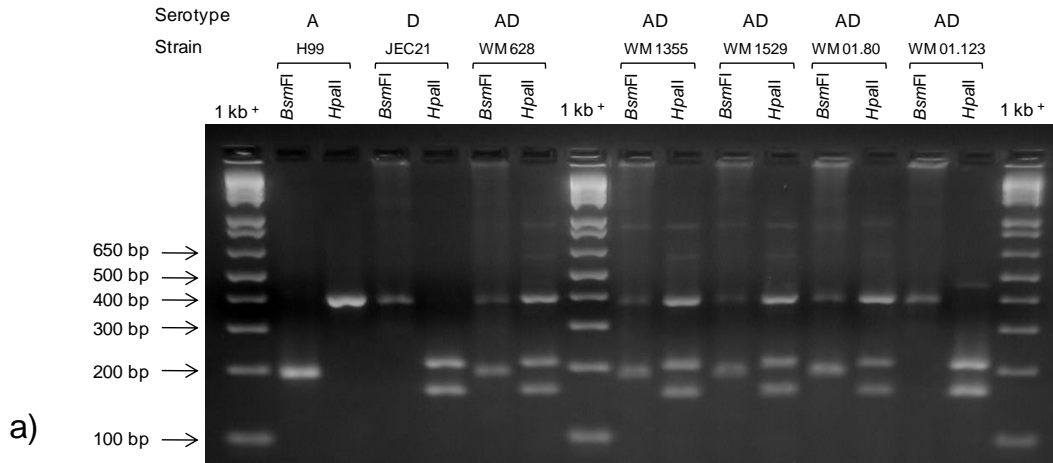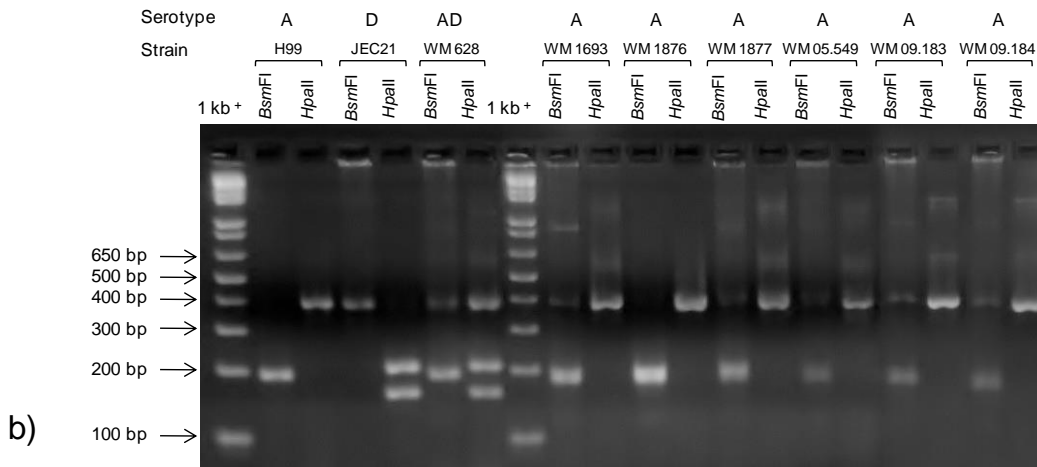

Supplement: S2 Fig — 1kb+ = DNA ladder (Invitrogen, Carlsbad, USA). (PDF) [file pone.0163955.s002.pdf]
