# Supplementary material for: Identification and Characterization of VNI/VNII and Novel VNII/VNIV Hybrids and Impact of Hybridization on Virulence and Antifungal Susceptibility Within the C. neoformans/C. gattii Species Complex
Source: PLoS One. 2016 Oct 20;11(10):e0163955. doi: 10.1371/journal.pone.0163955 (PMC5072701; doi:10.1371/journal.pone.0163955)
Supplement: S3 Fig — Bars denote the average of distribution and standard deviation. (PDF) [file pone.0163955.s003.pdf]

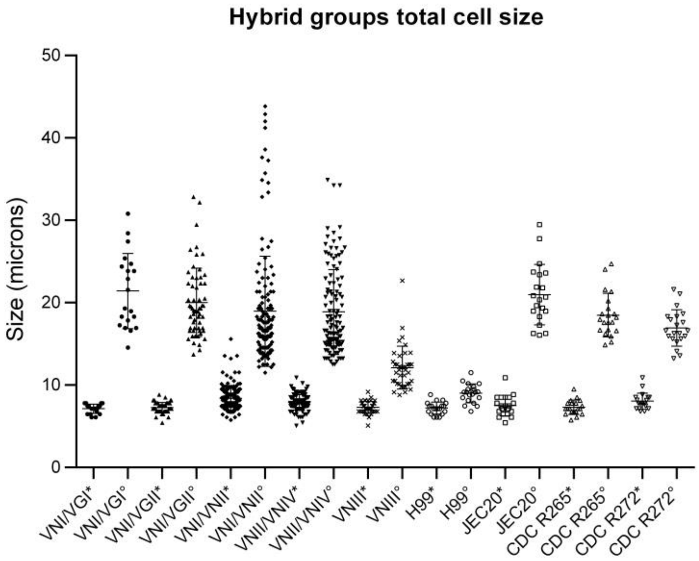

Hybrid groups total cell size
Size (microns)
VNI/VGI*
VNI/VGI°
VNI/VGII*
VNI/VGII°
VNI/VNII*
VNI/VNII°
VNII/VNIV*
VNII/VNIV°
VNIII*
VNIII°
H99*
H99°
JEC20*
JEC20°
CDC R265*
CDC R265°
CDC R272*
CDC R272°

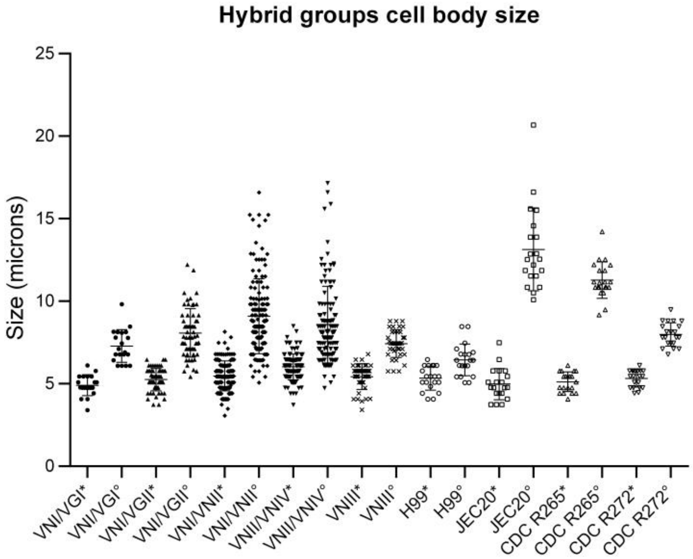

Hybrid groups cell body size
Size (microns)
VNI/VGI*
VNI/VGI°
VNI/VGII*
VNI/VGII°
VNI/VNII*
VNI/VNII°
VNII/VNIV*
VNII/VNIV°
VNIII*
VNIII°
H99*
H99°
JEC20*
JEC20°
CDC R265*
CDC R265°
CDC R272*
CDC R272°

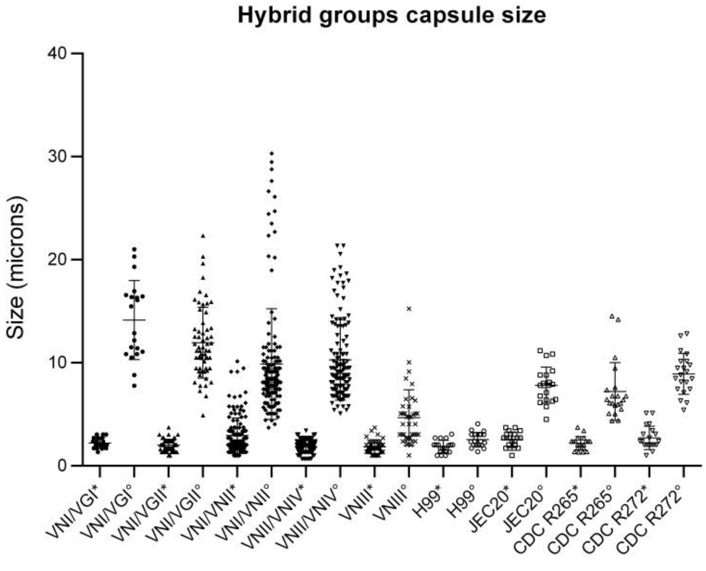

Hybrid groups capsule size
Size (microns)
VNI/VGI*
VNI/VGI°
VNI/VGII*
VNI/VGII°
VNI/VNII*
VNI/VNII°
VNII/VNIV*
VNII/VNIV°
VNIII*
VNIII°
H99*
H99°
JEC20*
JEC20°
CDC R265*
CDC R265°
CDC R272*
CDC R272°
