# Supplementary material for: Identification and Characterization of VNI/VNII and Novel VNII/VNIV Hybrids and Impact of Hybridization on Virulence and Antifungal Susceptibility Within the C. neoformans/C. gattii Species Complex
Source: PLoS One. 2016 Oct 20;11(10):e0163955. doi: 10.1371/journal.pone.0163955 (PMC5072701; doi:10.1371/journal.pone.0163955)
Supplement: S1 Table — (PDF) [file pone.0163955.s004.pdf]

S1 Table. PCR primer sequences.

Primer	Sequence (5'-3')	Reference
CH-Cap59F	CCTTGCCGAAGTTCGAAACG	[50]
CH-Cap59R	AATCGGTGGTTGGATTCAGTGT	[50]
JOHE7264	AGCTGATGCTGTGGATTGAATAC	[25]
JOHE7266	TGCAATCACAGCACCTTACATAG	[25]
JOHE7267	ATAGGCTGGTGCTGTGAATTAAG	[25]
JOHE7269	TGCAGTCACAGCACCTTCTATAC	[25]
JOHE7270	ATCAGAGACAGAGGAGCAAGAC	[25]
JOHE7271	CTAACTCTACTACACCTCACGG	[25]
JOHE7273	GTTCATCAGATACAGAGGAGTGG	[25]
JOHE7274	CTCAACTCTACTTCACCTCACAC	[25]
JOHE15634	GCTTGGCGTACGCTGTGG	[25]
JOHE15635	GGCGTCGCTTGGTACGGGT	[25]
JOHE15636	GGCGCGGCTTGGTAAGAGG	[25]
JOHE15629	GGTCCGCACTTGGGTAAGTG	[25]
JOHE15630	CTCACCCGCCTGAGTCTCAC	[25]
JOHE15631	GGCGTAGACGGACGAGCTC	[25]
JOHE3066	AATCTGCCCATCCAAACATTG	[25]
JOHE3236	GGCTATTTATCAATGGTTAGCGG	[25]
JOHE3065	AGTCGGCTATTTCTTATCGTC	[25]
JOHE2596	GCCAGAGAGATTCGATGTTG	[10]
JOHE3241	CATCGCTCCACATCTTCGTT	[10]
JOHE3240	TCCACCCCATTCATACCCG	[10]
STE12αF809	TTGACCTTTTRTTCCGCAATG	[17]
STE12αR1607	TTTCTTCTCCCCTGTTTATAGGC	[17]
STE12aF537	GTTCTTTGGAATGGCTTATTTCATAT	[17]
STE12aR1299	GMCTTGCGTGGATCATATCTA	[17]
MFαF	CTTCACTGCCATCTTCACCA	[49]
MFαR	GACACAAAGGGTCATGCCA	[49]
MFaF	CGCCTTCACTGCTACCTTCT	[49]
MFaR	AACGCAAGAGTAAGTCGGGC	[49]
