# Supplementary material for: Identification and Characterization of VNI/VNII and Novel VNII/VNIV Hybrids and Impact of Hybridization on Virulence and Antifungal Susceptibility Within the C. neoformans/C. gattii Species Complex
Source: PLoS One. 2016 Oct 20;11(10):e0163955. doi: 10.1371/journal.pone.0163955 (PMC5072701; doi:10.1371/journal.pone.0163955)
Supplement: S2 Table — (PDF) [file pone.0163955.s005.pdf]

S2 Table. Loci information outside the mating locus used for determination of α/α diploidization mechanism.

Locus	Product	Primer name and sequence	Amplification conditions	Reference
GPD1	Glyceraldehyde-3-phosphate dehydrogenase	GPD-f 5' ATGGTCGTCAAGGTTGGAAT 3′<br>GPD-r 5' GTA TTC GGC ACC AGC CTC A 3′	94 °C 3min; 35 cycles: 94 ºC 30s, 54 ºC 30s, 72 ºC 1min; 72 °C 10 min	[31]
PLB1	Phospholipase	PLB1F 5′ CTTCAGGCGGAGAGAGGTTT 3′<br>PLB1R 5′ GATTTGGCGTTGGTTTCAGT 3′	94 °C 3min; 30 cycles: 94 ºC 45s, 61 ºC 45s, 72 ºC 1min; 72 °C 10 min	[13]
SOD1	Cu, Zn superoxide dismutase	SOD1-f 5′TCTAATCGAAATGGTCAAGG 3′<br>SOD1-r 5′ CGCAGCTGTTCGTCTGGATA 3′	94 °C 3min; 35 cycles: 94 ºC 30s, 52 ºC 30s, 72 ºC 1min; 72 °C 10 min	[13]
URA5	Orotidine monophosphate pyrophosphorylase	URA5F 5′ ATGTCCTCCCAAGCCCTCGAC 3′<br>URA5R 5′ TTAAGACCTCTGAACACCGTACTC 3′	94 °C 3min; 35 cycles: 94 ºC 45s, 63 ºC 45s, 72 ºC 1min; 72 ºC 10min	[4]
IGS1	Ribosomal RNA intergenic spacer	IGSF 5′ ATCCTTTGCAGACGACTTGA 3′<br>IGSR 5′ GTGATCAGTGCATTGCATGA 3′	94 °C 3min; 35 cycles: 94 ºC 30s, 60 ºC 30s, 72 ºC 1min; 72 ºC 10min	[13]
