# Supplementary material for: Identification and Characterization of VNI/VNII and Novel VNII/VNIV Hybrids and Impact of Hybridization on Virulence and Antifungal Susceptibility Within the C. neoformans/C. gattii Species Complex
Source: PLoS One. 2016 Oct 20;11(10):e0163955. doi: 10.1371/journal.pone.0163955 (PMC5072701; doi:10.1371/journal.pone.0163955)
Supplement: S3 Table — (PDF) [file pone.0163955.s006.pdf]

S3 Table. Mating type and serotype- and mating-type-specific PCR analysis results for the VNI/VNII hybrid isolates in comparison with reference strains.

Trait	Gene	Strain										
		**WM 714**	**WM 1986**	**WM 2059**	**WM 2064**	**WM 2068**	**WM 2059**	**WM 05.269**	**H99 (Aα)**	**IUM 96-2828 (Aa)**	**JEC21 (Dα)**	**JEC20 (Da)**
**Genes in the *MAT* locus**	*STE20Aα*	+	+	+	+	+	+	+	+	–	–	–
	SXI1Aα	+	+	+	+	+	+	+	+	–	–	–
	STE20Dα	–	–	–	–	–	–	–	–	–	+	–
	SXI1Dα	–	–	–	–	–	–	–	–	–	+	–
	*STE20A***a**	–	–	–	–	–	–	–	–	+	–	–
	*SXI2A***a**	–	–	–	–	–	–	–	–	+	–	–
	*STE20D***a**	–	–	–	–	–	–	–	–	–	–	+
	*SXI2D***a**	–	–	–	–	–	–	–	–	–	–	+
	STE12α	+	+	+	+	+	+	+	+	–	ND	ND
	*STE12***a**	–	–	–	–	–	–	–	–	+	ND	ND
**Other genes**	*GPA1*-A	+	+	+	+	+	+	+	+	+	–	–
	PAK1-A	+	+	+	+	+	+	+	+	+	–	–
	GPA1-D	–	–	–	–	–	–	–	–	–	+	+
	PAK1-D	–	–	–	–	–	–	–	–	–	+	+

ND: not done
