# Supplementary material for: Identification and Characterization of VNI/VNII and Novel VNII/VNIV Hybrids and Impact of Hybridization on Virulence and Antifungal Susceptibility Within the C. neoformans/C. gattii Species Complex
Source: PLoS One. 2016 Oct 20;11(10):e0163955. doi: 10.1371/journal.pone.0163955 (PMC5072701; doi:10.1371/journal.pone.0163955)
Supplement: S4 Table — (PDF) [file pone.0163955.s007.pdf]

S4 Table. Cumulative survival of larvae for each hybrid group and control strains.

**Hybrid groups/control strains**	**Mating type allelic pattern**	**Mortality on day 14 post inoculation (%)**	**Standard error (%)**	**Median survival**	**Standard error**
VNI/VGI	αABa	100	-	5	-
VNI/VGII	αABa	100	-	4	0.17
VNI/VNII	αAAα	86.4	2.9	4	0.29
VNII/VNIV	aADα	30.8	4.2	>14	-
VNIII	αADa	100	-	6	0.05
CDC R265	Bα	100	-	5	0.15
CDC R272	Bα	100	-	7	0.27
H99	Aα	100	-	4	0.34
JEC 20	Da	20	8.9	>14	-
