# Supplementary material for: Identification and Characterization of VNI/VNII and Novel VNII/VNIV Hybrids and Impact of Hybridization on Virulence and Antifungal Susceptibility Within the C. neoformans/C. gattii Species Complex
Source: PLoS One. 2016 Oct 20;11(10):e0163955. doi: 10.1371/journal.pone.0163955 (PMC5072701; doi:10.1371/journal.pone.0163955)
Supplement: S5 Table — (PDF) [file pone.0163955.s008.pdf]

S5 Table. Summary of previously published data of MICs (μg/ml) range and geometric mean (GM) values of 5-Flucytosine and Amphotericin B agents against different cryptococcal strain groups.

Strain group	MIC Parameter	MICs (μg/ml)		Reference
		5-Flucytosine	Amphotericin B	
VNI	Range	1-16	0.25-2	[87]
	GM	3.43	0.56	[87]
VGI	Range	1-8	0.25-1	[87]
		0.5-4	0.25-0.5	[86]
		0.125-16	<0.016-0.5	[82]
	GM	2.13	0.47	[87]
		1.16	0.36	[86]
		1.667	0.238	[82]
VGII	Range	0.25->64	0.25-2	[87]
		2-8	0.25-0.5	[86]
		0.5->64	0.125-1	[82]
	GM	4	0.62	[87]
		3.81	0.39	[86]
		5.033	0.312	[82]
Serotype A	Range	1-8	0.06-0.5	[84]
	GM	3.123	0.25	[84]
Serotype D	Range	0.5-8	0.03-1	[84]
	GM	3.204	0.156	[84]
Serotype AD	Range	0.25-16	0.06-0.5	[84]
	GM	4.443	0.174	[84]
Serotype B	Range	0.06-16	0.125-1	[84]
	GM	1.66	0.239	[84]
C. neoformans	Range	2-4	0.13-0.5	[86]
		0.25-16	0.03-1	[84]
		2-16	0.25-2	
	GM	2.97	0.31	[86]
		3.6	0.186	[84]
		5.12	0.51	
C. gattii	Range	0.5-8	0.13-0.5	[86]
		0.06-16	0.125-1	[84]
		0.125->64	<0.016-1	[82]
		0.5->64	0.25-2	[83]
	GM	2.19	0.35	[86]
		1.97	0.24	[84]
		2.896	0.272	[82]
		6.16	0.59	[83]
